# Remote Sensing Data Reveal a Significant Reduction in the Area of the Nesting Habitat of *Rafetus euphraticus* in the Tigris River, Southeastern Turkey

**DOI:** 10.1002/ece3.70691

**Published:** 2024-12-16

**Authors:** Murat Biricik, Kamran Safi, Şemsettin Turğa

**Affiliations:** ^1^ Department of Migration Max‐Planck Institute of Animal Behavior Radolfzell Germany; ^2^ Department of Biology University of Konstanz Konstanz Germany; ^3^ Fabrika Mah. 262. Sokak Hayatkent‐1 Sitesi Diyarbakır Turkey

**Keywords:** conservation, dam, Euphrates Softshell Turtle, habitat suitability, sand mining

## Abstract

The Euphrates Softshell Turtle ( *Rafetus euphraticus* ) is an endangered freshwater turtle native to the Tigris‐Euphrates river system. Habitat destruction caused by dams and sand mining poses a major threat to the species. This study quantitatively assesses the occurrence of sandy areas in the upper Tigris in Turkey as a key component of their nesting habitat, utilizing remote sensing data. An ensemble approach was employed, applying and combining Generalized Additive Models (GAM), Generalized Boosting Models (GBM), Generalized Linear Models (GLM), and Random Forests (RF) for classification. The models indicate that, compared to 2018, the occurrence of sandbanks—assumed to be crucial nesting habitat for this species—was reduced by 41% in 2022 following the construction of a large dam. Additionally, sand mining and flooding of adjacent lands have fragmented and degraded the remaining sandbanks. The survival of the Turkish population of the Euphrates Softshell Turtle requires immediate and effective action.

## Graphical abstract

The Euphrates Softshell Turtle ( *Rafetus euphraticus* ) is an endangered freshwater turtle native to the Tigris‐Euphrates river system. As a key component of their nesting habitat, this study quantitatively assesses the occurrence of sand areas in the upper part of the Tigris in Turkey based on remote sensing data. Based on an ensemble approach, several models were applied and combined for classification. The models suggest that compared to 2018 the occurrence of sandbanks, which are crucial for reproduction of this species, were reduced by 41% in 2022 after the construction of a big dam. Although the river shoreline, potentially accessible to the turtles increased by about 1.52 times, the newly gained shores hardly provide habitat for reproduction. The remaining sand areas have become smaller, are highly fragmented and degraded. The survival of the Turkish population of the Euphrates Softshell Turtle requires immediate effective action.

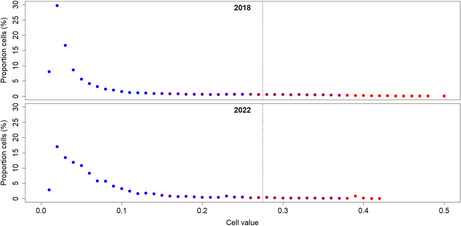

## 1 Introduction

Several streams worldwide, especially in North America, Asia and Australia, have degraded by siltation, river impoundments, and sand mining (Stanford et al. 2020). Adverse impacts on rivers typically occur in areas with low rainfall and high population, as well as in areas with intensive agricultural or industrial activity, where dams and artificial channels are used for agricultural irrigation (Mostafavi et al. 2021). In Turkey, one of the largest regional development projects in the world planned to build 22 dams, 19 hydroelectric power plants, and extensive irrigation networks to utilize available water resources of Southeastern Anatolia (Bilgen et al. 2021).

Of all the 360 Testudine species, 187 (51.9%) are considered Threatened (in categories VU, EN, or CR) by IUCN Red List criteria, and of these, 127 species (67.9%) are Endangered or Critically Endangered. Habitat loss, degradation and fragmentation affect 80% of the 25 species of the world's rarest turtles and tortoises (Stanford et al. 2020). Habitat quality is a key factor in the conservation of aquatic or semi‐aquatic turtle species. Freshwater turtles are worldwide at direct and indirect threat of extinction due to changes caused by damming leading to the loss of terrestrial and aquatic nesting habitats (Stanford et al. 2020), changes to food availability, and blocking movement (Bárcenas‐García et al. 2022). Dams change running water sections of rivers to still water pools with a profound effect on the ecosystems, changing sediment and nutrients propagation downstream, altering fluvial thermal regimes, and disconnecting river segments (Mostafavi et al. 2021). Dam construction harms turtle populations not only by interrupting migratory routes and disrupting gene flow but also by deteriorating habitat, particularly nesting sites (Bodie 2001). Dams prevent upstream sediment from replacing the sand deposits lost by flows (Moll and Moll 2004; Lenhart, Naber, and Nieber 2013; Le Duc et al. 2020). Aggravating the detrimental effects of dams, mining and exploiting existing sand reserves damages fauna at significant levels (Leal Filho et al. 2021) through removing fine sand from the fluvial areas. Several turtle species require a water flow below a certain level for nesting, and river species are particularly affected by hydrological regimes. Immediate changes in the flow can affect the availability of different in‐stream habitat features for turtles. Turtles need dropping water levels during the summer to avoid flooding their nests since most eggs cannot survive submersion for more than two days (Lenhart, Naber, and Nieber 2013).

**FIGURE 1:**
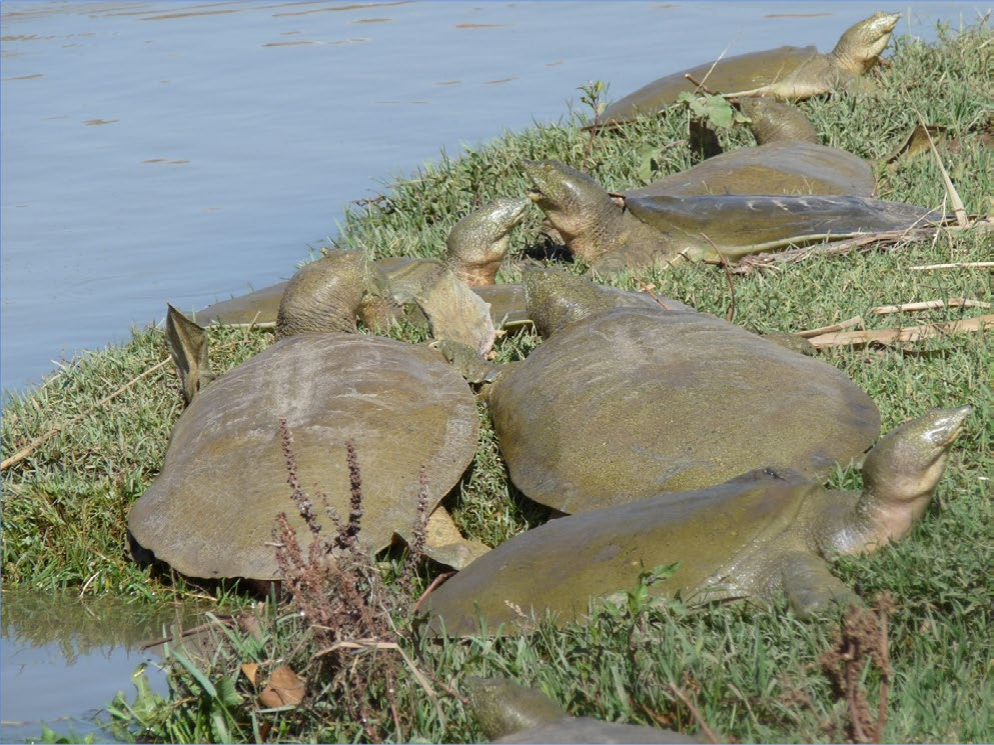
Euphrates Softshell Turtles ( *Rafetus euphraticus* ) basking on the banks of the Tigris River.

**FIGURE 2:**
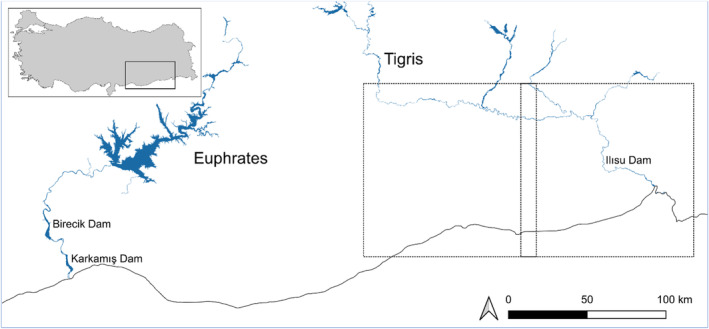
Map of the upper parts of the Tigris and Euphrates Rivers in Turkey (inset), along with the study area (solid lines) which is restricted to the main channel and major tributaries of the Tigris River delineated by two overlapping adjacent Sentinel‐2 image tiles (dashed lines, see text). The localities of dams mentioned in the text were also shown.

The Euphrates Softshell Turtle ( *Rafetus euphraticus* ) is a freshwater turtle endemic to the Tigris‐Euphrates river system, currently listed as Endangered by the IUCN (Ghaffari et al. 2017; Figure 1). Unsustainable management and use of the basin's hydrological system, primarily through the use of dams and the presence of sand quarries, is greatly impacting this ecosystem and consequently the Euphrates Softshell Turtle (Gramentz 1993; Biricik and Turğa 2011; OSB 2015; Ghaffari et al. 2017; Bachmann et al. 2019). On the Turkish part of the Euphrates (Figure 2), five large dams, ranging from 157 hm^3^ to 48,700 hm^3^ in reservoir capacity and 28.4–817 km^2^ in reservoir surface area, have been constructed between 1974 and 2000 (GAP 2020). This progress impacted the Euphrates Softshell Turtle heavily (Gramentz 1993), causing loss of suitable habitats and declining population levels in the Euphrates (Taşkavak and Atatür 1998).

The Yangtze Giant Softshell Turtle ( *Rafetus swinhoei* ), taxonomically the only remaining sister species to *R. euphraticus* in the genus (Le et al. 2014) was represented by just three individuals known to have survived (Fong et al. 2021). In Vietnam, sandy riparian beaches along rivers are the most important breeding habitat for this species, where the dramatic decline in recent sightings suggests a population collapse associated, among other factors, with damming, despite the low comparability of records in the periods before and after the construction of the suspected dams (Le Duc et al. 2020).

The upper Tigris basin constitutes the northernmost distribution of *R. euphraticus* (Taşkavak and Atatür 1998). This area lies within the Irano‐Anatolian Biodiversity Hotspot, constituting an extensive and until recently intact river ecosystem with islands, bays, and sandbanks. Recent dramatic changes comparable to the Euphrates basin threaten this hotspot (Bachmann et al. 2019). Since 1998, five energy dams have already been built in the Tigris basin, and another three are under construction (GAP 2020). The largest and most impactful is the Ilısu Dam (Figure 2), which has a catchment area of 309 km^2^ and a reservoir capacity of 10,600 hm^3^. The project resulted in permanent loss of the natural riparian ecosystem and other associated habitats of the Tigris river course for over 170 km (Biricik and Karakaş 2012).

With the beginning of water retention by Ilısu Dam on the Tigris in 2019, large amounts of *R. euphraticus* nesting places must have been irreversibly lost to rising water. This incident has previously occurred at nesting sites in the Euphrates: Taşkavak and Atatür (1998) assessed the existence of suitable nesting sites on the banks of the dam reservoir as highly doubtful. They stressed that natural sandbars are being lost as daily fluctuations in water levels from dams complicate the establishment of nesting sites downstream. An estimated 400 km of the riverbanks of the Tigris and its tributaries are currently affected upstream of the Ilısu Dam, turning a dynamic river system into a static reservoir. The downstream ecosystem is also likely to be impacted by water surges caused by the operation of the power plant (Bachmann et al. 2019).

Our knowledge of the natural nesting behavior of the Euphrates Softshell Turtle is limited. We do not know whether the species exhibits communal nesting at specific nest sites, such as many river turtles have been found to do (Moll and Moll 2004), or whether nests are laid more generally along the river. Field data to characterize nest site selection are scarce and incidental. Taşkavak and Atatür (1998) found two nests, both buried into fine sand on the banks of the Euphrates. The authors described ideal nesting areas as wide sandy areas, sandy riverbanks, or large and small islets in the middle of the river. Biricik and Turğa (2011) discovered a nest chamber along the Tigris which contained fresh laid eggs in fine sand. Additional observations also suggested that sand constituted an important component of the nesting places of the species (Ghaffari et al. 2013; Fazaa, Dunn, and Whittingham 2015; Bayrakçı 2023).

As a potentially crucial component of the habitat, we focus here on the availability of fine sand (with an approximate grain size < 0.4 mm; *see* Biricik and Turğa 2011) zones to the Euphrates Softshell Turtle for nesting in the Tigris area. Novel approaches based on remotely sensed data and the machine learning techniques provide the potential for specifying and monitoring airborne ground grain size along rivers (Kryniecka and Magnuszewski 2020; Gómez et al. 2022; Marchetti et al. 2022). In these works, the particle size classes of the substrate were determined using spectral predictors in their images. Here we use a corresponding approximation of modeling that approaches the task of identifying sandy areas across the potential habitats using information obtained from labeled sandbar zones. Considering the apparent lack of field data and the adversity and the expenses associated with obtaining this data, the method represents a valuable alternative. This study models Euphrates Softshell Turtle nesting habitat changes during the most recent years in the upper (Turkish) part of the Tigris River, based on satellite sensing data to classify sand zones as a predictor of nesting areas.

Bayrakçı, Ayaz, and Luiselli (2021) evaluated the habitat change in the Euphrates using GIS techniques, and concluded that areas potentially available to the Euphrates Softshell Turtle, such as water bodies and shoreline, increased by more than 100% between the 1990s and 2018, while appraising an increase of 413% in the “preferred turtle habitat types”, ignoring the availability of sandbanks. To compare the corresponding alterations in the Tigris area, we also applied the methodology described in this publication, and discussed the results.

## 2 Methods

### 2.1 Study Area

**FIGURE 3:**
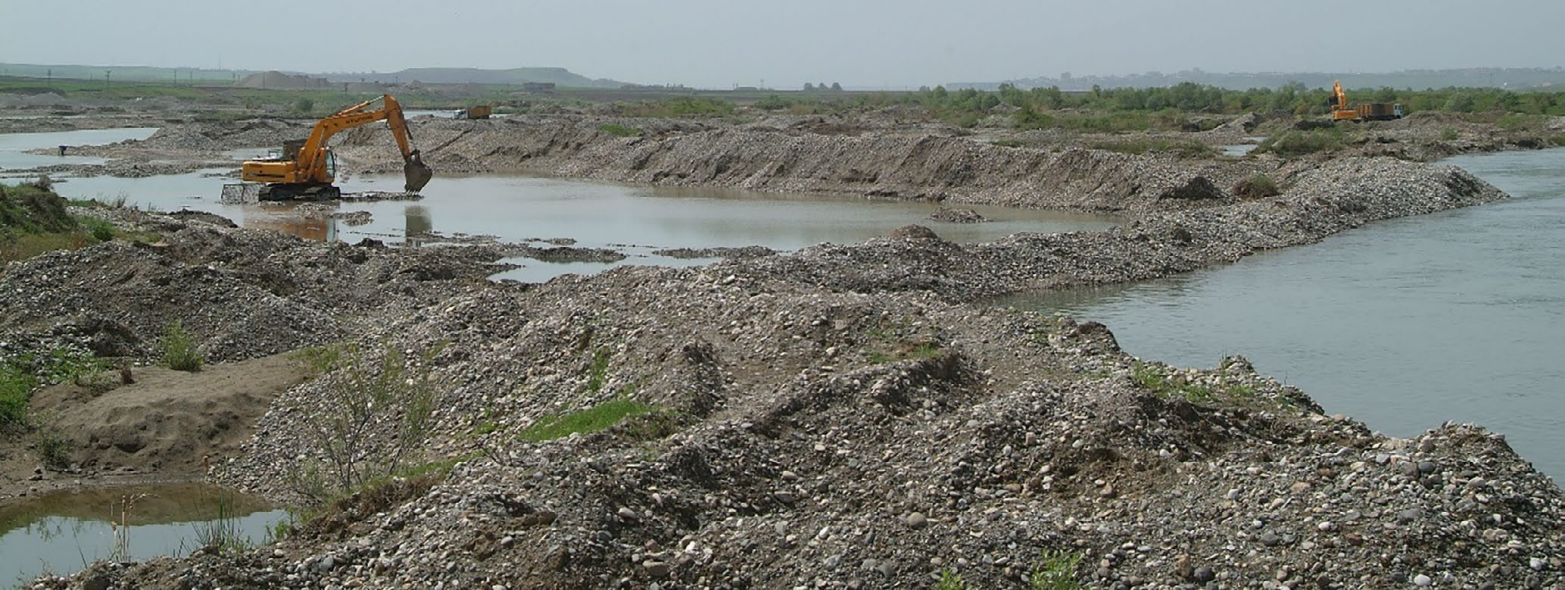
Sand quarries in the large areas in the upper part of the Tigris causing severe habitat destruction.

The Tigris River originates in the Eastern Turkey and flows southward from an elevation of 900 m above sea level (a.s.l.) in the southeastern Taurus Mountains to 340 m a.s.l. at the Syrian‐Iraqi border (Figure 2). The river is about 1900 km long and traverses a stretch of 523 km through Turkey. Crop fields are widespread throughout the region, and both dry and irrigated agriculture are intensively practiced in the areas adjacent to the river. Poplar farming is carried out on suitable lands. Natural woody vegetation along the river mainly consists of *Tamarix* sp., *Salix* sp., and *Populus euphratica*. Remnants of reedbeds around the ponds and marshes are also found. Sandbanks along the river provide suitable nesting habitat for the Euphrates Softshell Turtle (Biricik and Turğa 2011; Bachmann et al. 2019). Current riverine habitats are severely degraded by intense agricultural usage and especially sand mining activities, which has increased significantly over the last three to four decades due to intensifying urbanization and the need for building material (personal observation; Figure 3). The region has a steppe climate characterized by cold winters with scarce precipitation, and hot and arid summers (Biricik and Karakaş 2012).

The chosen study area did not cover the entire known distribution of the species in the Turkish part of the Tigris (Taşkavak and Atatür 1998; recent observations of our own), ignoring a small part in the very north of the species' distribution area which corresponds a latitudinal distance of 26 km. This served to simplify the work, as the species was detected there only in very small numbers and also in areas that have already been heavily modified (Figure 2).

### 2.2 Acquisition of Sentinel‐2 Images

Satellite images were downloaded from the Copernicus Open Access Hub browser (scihub.copernicus.eu). We selected Level‐2A Sentinel‐2 data, which provide corrected reflectance orthoimages derived from the associated (Level‐1C) data after applying an atmospheric correction. The images are composed of 100 × 100 km^2^ tiles in UTM/WGS84 projection (sentinels.copernicus.eu/web/sentinel/missions/sentinel‐2). Covering the entire study area requires two Sentinel‐2 image tiles (with the identification numbers T37SFB and T37SGB). We looked for images with no or minimal (< 10%) cloud coverage, corresponding to sensing dates in summer 2018 and 2022, when water levels are lowest. We used images from July 19, 2018 and July 28, 2022 to compare the study area before and after the commissioning of the Ilısu Dam in 2019.

We compared rainfall amounts for the two respective sensing dates of the images to assess whether river shorelines may have been affected between the 2 years due to differences in precipitation. Precipitation data measured by the Diyarbakır Meteorological Station (located on the upper Tigris) between October and June (comprising the annual rainy season in the region) prior to the two dates were used (data retrieved from www.visualcrossing.com). No significant change in monthly average precipitation was detected (Wilcoxon signed‐rank test, *n* = 9, *Z* = 10, *p* > 0.1). Therefore, we assumed that precipitation was not responsible for any change in the shore line areas in July 2018 and 2022.

### 2.3 Spatial Data Processing

To obtain a single multiband raster image of the river at 10 m resolution, we merged two same‐date Sentinel‐2 tiles from both sides of the river for each date. To map the Tigris River area in 2018 and 2022 respectively, we retrieved the Normalized Difference Water Index (NDWI) from Sentinel‐2 satellite image (McFeeters 1996). The Sentinel Water Index (SWI) is an alternative option (Jiang et al. 2021) that outperforms NDWI in terms of distinguishing water bodies from several types of land surfaces such as urban areas, sediment, and silt (Jiang et al. 2021; Goswami and Ghosal 2022). However, the highest resolution available under this method is 20 m due to the sensitive characteristic bands it uses. Therefore, some narrow rivers and small lakes cannot be accurately extracted, particularly in urban areas due to the complex background and building shadows. On the other hand, NDWI is also prone to missing small waterbodies as it confuses them with other land cover types (Jiang et al. 2021; Saghafi, Ahmadi, and Bigdeli 2021). Considering the pros and cons, we performed both of the methods, and determined that NDWI had conveniently delineated the main river channel. Nevertheless, a minor manual refinement process was required later, particularly around urban areas on the shore.

A 100 m buffer surrounding the river course was produced and vectorized to delimit the riparian zone (Bodie 2001). Normalized Difference Vegetation Index (NDVI) was calculated using near infrared (NIR) and red bands of the Sentinel‐2 images (Band 8 and Band 4, respectively; www.earthdata.nasa.gov/sensors/sentinel‐2‐msi). We established NDVI‐threshold for vegetation as 0.2 (Wong, Fung, and Yeung 2019). After a binary classification, raster cells indicating vegetated areas were excluded as these were assumed as unsuitable for nesting of the study species. We cropped Sentinel‐2 images according to the study area's limits and masked them by the river course including the riparian zone. The cells corresponding to non‐water and non‐vegetated parts inside the cropped area were considered as “potentially suitable nesting habitat” (Merow et al. 2022), which delimited the study area in focus.

The tiles of Sentinel‐2 time series can deviate in terms of reflectance values due to different conditions on different sensing days (Henriques et al. 2022). To intercalibrate the images, we used each band of 2018 images as reference and then we calculated band‐wise major axis regressions with the corresponding bands of the 2022 images, which were then corrected using the regression coefficients calculated (Granadeiro et al. 2021).

### 2.4 Presence/Absence Data

A total of 171 point coordinates on sandbanks in the study area were determined using open Google Earth historical satellite images based on the expert knowledge of one of the authors (MB) who conducted several fieldwork initiatives in the area during last decades. The images used were mostly dated in July 2018 whenever available, or during the adjacent earlier or later months. The sandbanks chosen had a minimum surface area of about 500 m^2^ and were located on either shores of the river or on large islets.

As background data, a total of 2000 random points were generated throughout the potentially suitable nesting habitat area. The minimum distance we allowed between any (sand or background) point was about 77 m to avoid locations to fall on the same raster cell. Presence and absence point coordinates used in this study are available as Table S1 and Figures S1 and S2.

### 2.5 Data Analysis and Modeling

In order to distinguish the sand zones, we used a supervised classification approach. The dependent variable in the model was substrate type, namely sand versus non‐sand areas, assigned to a given raster cell, and the explanatory variables were values of Sentinel‐2 bands (Band 1 through Band 12) that were acquired for the corresponding cell. Band 10 was omitted, as this is used for the cirrus correction and does not contain surface information (sentinels.copernicus.eu).

We calculated the variance inflation factor (VIF) to detect any correlation between variables (Guisan et al. 2017). Highly correlated variables above the threshold of *r* = 0.7 were removed. After the exclusion of the correlated bands from the dataset, a total of five Sentinel‐2 bands were determined as the most informative and entered into the model (Table 1). The sampling (presence/absence) coordinates were also added to the models to deal with any spatial autocorrelation (Dormann et al. 2007).

**TABLE 1:** Sentinel‐2 bands used in the models.

Band	Resolution (m)	Central wavelength (nm)	Description
Band 2	10	490	Blue
Band 7	20	783	Visible and near infrared (VNIR)
Band 8	10	842	Visible and near infrared (VNIR)
Band 9	60	940	Short wave infrared (SWIR)
Band 12	20	2190	Short wave infrared (SWIR)

We implemented an ensemble approach, widely used in species distribution modeling (SDM), to predict the presence of sand zones throughout the potentially suitable nesting habitats in 2018. Four algorithms were used: Generalized Additive Model (GAM), Generalized Boosting Model (GBM), Generalized Linear Model (GLM), and Random Forest (RF). A ratio of 0.7/0.3 was used to randomly split the data into a training set for model building and an evaluation set to determine its performance. We repeated the split sample 20 times to account for uncertainty. The contribution of each predictor variable of the classification models was assessed using related functions of the statistical package used. To evaluate the models' performance, we used area under the receiver operating characteristic curve (AUC) with a binomial distribution. We used all the models, as they showed good performance (*see* Results), to create ensemble models that weighted single models by their model performance of true skill statistic (TSS). Two ensemble models were created based of the remote sensing data from 2018 and 2022 as reference and predicted models, respectively. AUC was also calculated to determine the reference ensemble model performance. Probability prediction maps with a resolution of 10 m were produced for potentially suitable nesting habitats, and the accuracy assessments were also made by visual comparison of the maps with the images from Google Earth.

The frequencies of cell values on the produced raster maps were rounded to the nearest 0.01, and were ranked by equal intervals into six categories between minimum and maximum values obtained, which were designated as very low (0–0.1) to best (0.5–0.6) in terms of the assumed habitat quality. For the binary classification of sand areas in the potential habitat, we chose the mean of the predicted probabilities of the cell values as threshold as recommended by Liu et al. (2005). A Mann–Whitney‐Wilcoxon test was implemented to the categories for the determination of differences between the 2018 and 2022 predictions.

### 2.6 Software

We used QGIS (version 3.32.0) to visualize and analyze spatial data (QGIS Development Team 2023). For the analyses, the *R* software (version 4.2.3; R Core Team, 2024) was used. Modeling procedures were implemented by using the package “*sdm*” along with its dependents (version 1.1‐8; Naimi and Araújo 2016).

## 3 Results

### 3.1 Model Evaluation

The evaluation showed that the models had good performances with AUC between 0.83 and 0.88. Random Forest algorithm proved to be the best performing model (Table 2).

**TABLE 2:** Model mean performances using test dataset (with bootstrap and subsampling data partitioning, 20 replicates for each algorithm, and 30% test percentage).

Methods	AUC	COR	TSS	Deviance
GAM	0.85	0.39	0.62	0.44
GBM	0.85	0.43	0.61	0.44
GLM	0.83	0.28	0.58	0.47
Random Forest	0.88	0.54	0.66	0.37

The response curves suggest that Bands 2 and 7 had a positive relationship, while Bands 8 and 9 had a negative relationship with probability of sandiness (S1). Sentinel‐2 Bands 2 and 8 were the most important predictors of sand zones in the study area (S2). The reference (2018) ensemble model performed very well with an AUC of 0.95.

### 3.2 Changes in Sand Areas

**FIGURE 4:**
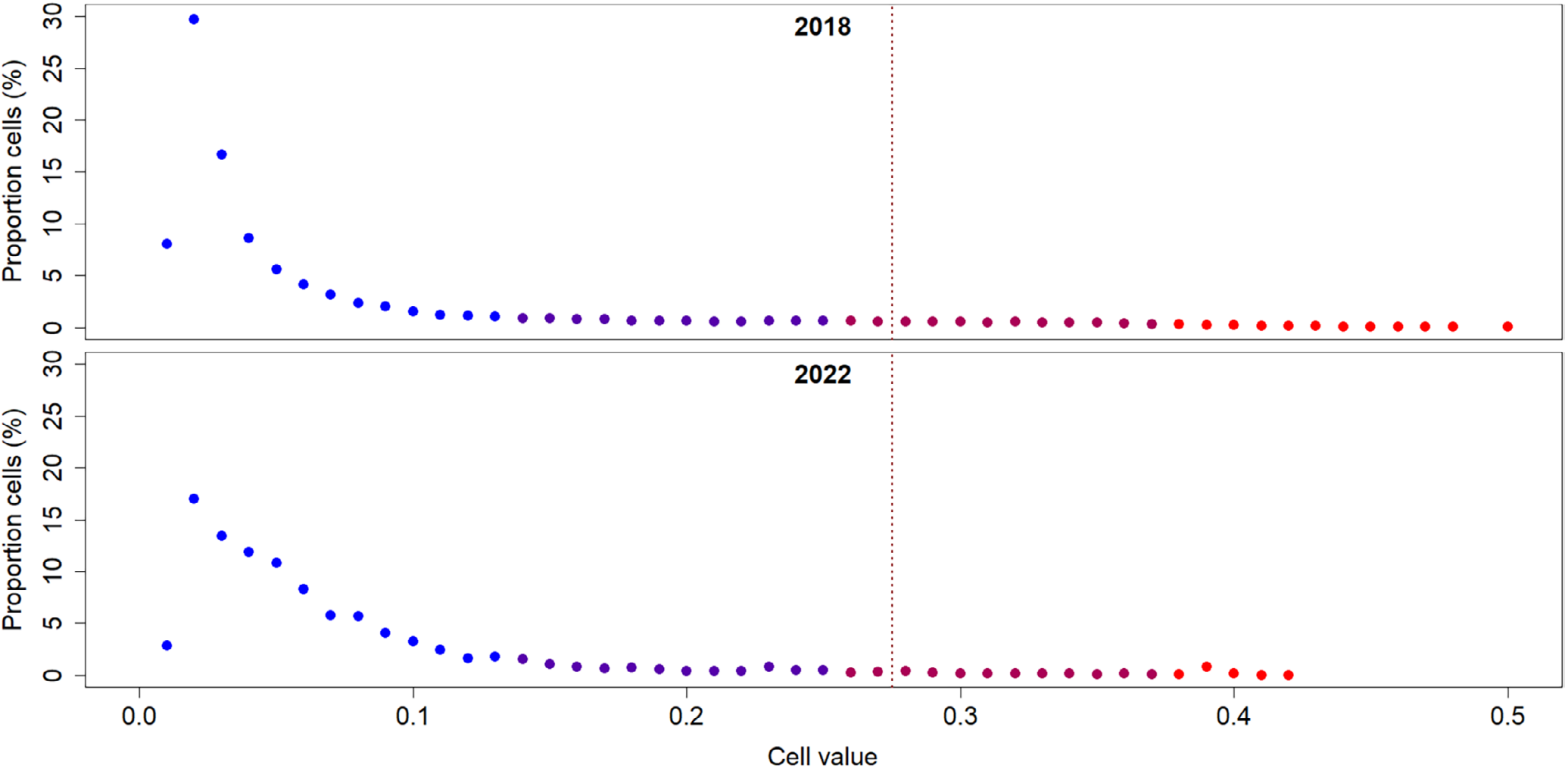
Percentual distribution of grouped cell values by years in the ensemble model raster maps indicating quality loss in the nesting habitats of *Rafetus euphraticus* in the upper part of the Tigris. The probability of sand habitats is represented by blue and red dots, with blue indicating lower probability and red indicating higher probability. The sand habitats are mainly separated by the threshold, which is shown by the vertical dashed line.

Quantitative and qualitative comparisons of the ensemble model raster map cell compositions for 2018 and 2022 show that the number of raster cells throughout the potentially suitable nesting habitat area of *Rafetus euphraticus* increased by 1.52 times in total (Table 3). Also, the mean predicted cell values slightly increased from 0.0749 ± 0.00027 (mean ± SE) to 0.0753 ± 0.00017 (*p* < 0.001, Mann–Whitney‐*U*‐test). However, the number of cells with values above 0.2 (classes 3–6), which were considered as suitably good quality habitat areas due to the higher probability of sand components, decreased from 10.7% to 6.0% of the corresponding total cell numbers (Table 3, Figure 4).

**TABLE 3:** Areal coverage of the habitat quality in terms of the ensemble model raster cells (numbers have been rounded).

Cell classes	2018			2022			Change (2022 − 2018)		
	No. cells	%	Total cell values	No. cells	%	Total cell values	No. cells	%	Total cell values
1	83,609	80.1	3160	126,822	79.7	6210	43,213	−0.4	3050
2	9690	9.3	1378	22,849	14.4	3070	13,159	5.1	1692
3	6106	5.9	1523	6176	3.9	1511	70	−2	−12
4	4258	4.1	1461	2947	1.9	1077	−1311	−2.2	−384
5	697	0.7	296	286	0.2	116	−411	−0.5	−181
6	6	0	3	0	0	0	−6	0	−3
Total	104,366	100	7821	159,080	100	11,984	54,714	0	4162

*Note:* Cell class 1 represents the lowest probability of sand habitats in the study area, while cell class 6 represents the highest probability.

**FIGURE 5:**
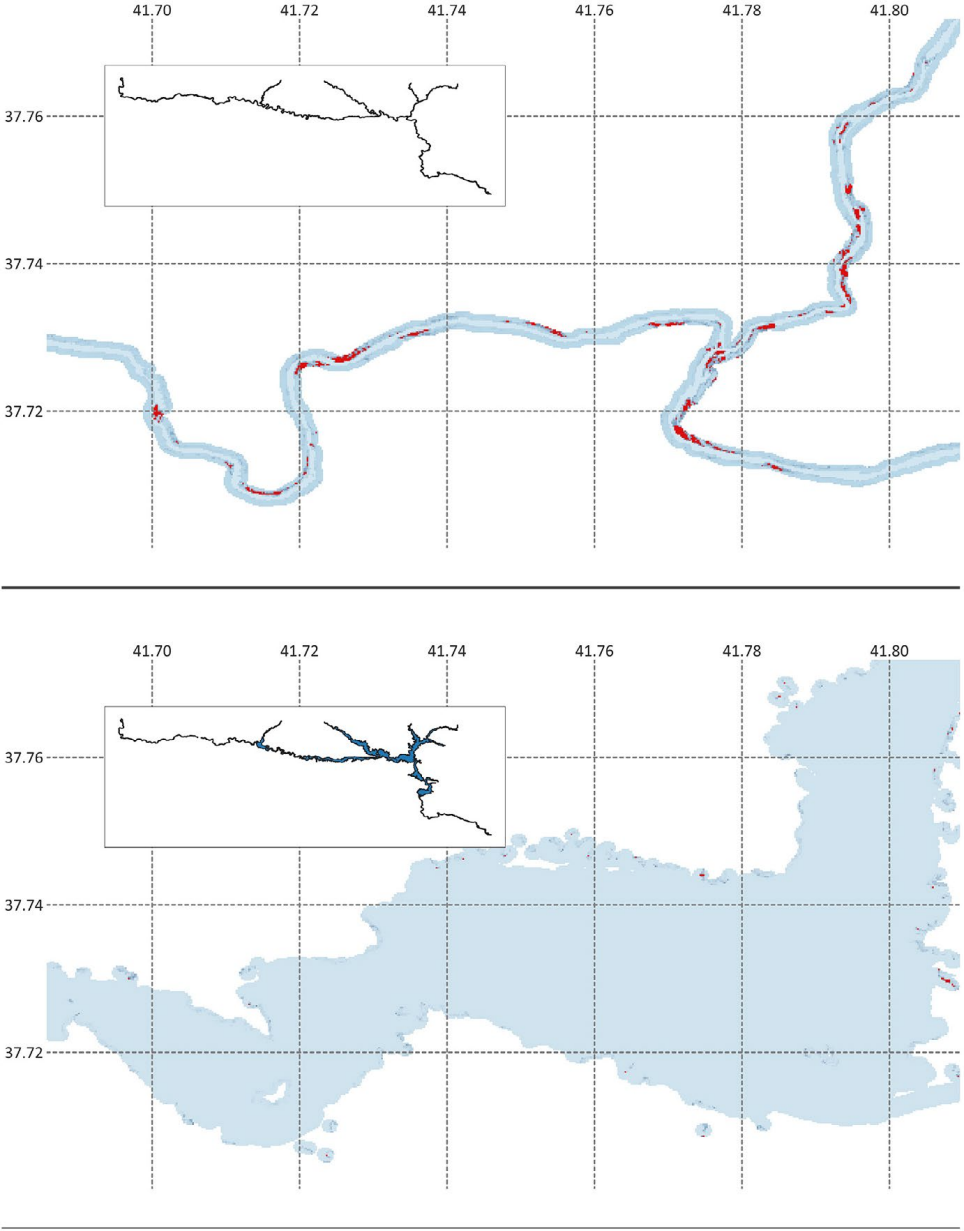
An example location indicating dramatic disappearances of sand banks (red patches) on the potential nesting zone (dark blue coastal areas) before (top panel) and after (bottom panel) the water retention by the Ilısu Dam in the upper Tigris area (insets).

An evaluation of the distribution of the cells with relatively high values on the maps illustrates the disappearance of significant sandbanks in favor of the highly dispersed small patches of sandy areas. The expansion of the shoreline due to inundations of valleys and arising of former hills as new islets clearly increased the total surface area and complexity of the shores without much or any contribution of suitable habitats for the species (Figure 5).

Furthermore, we implemented the methodology described in Bayrakçı, Ayaz, and Luiselli (2021) to the Tigris area in order to illustrate the change that occurred between 2018 and 2022. We used CORINE Land Cover (CLC) 2018 data to identify the areas of CLC classes that were classified as “turtle habitat types” in the publication. The results show that the water course buffer area has increased by 21%, while the total area of “turtle habitat types” has decreased by 32.78% over the years considered here. CLC class “permanently irrigated land” constituted about 99% and 98% of the total “turtle habitat types” in 2018 and 2022, respectively (Table 4).

**TABLE 4:** Change in surface (polygon) areas (as hectare and percentage of total water course buffering area) of Corine Land Cover (CLC) 2018 classes determined in the study area (Tigris) during the years studied.

CLC classes	2018		2022	
Beaches, dunes, sands	1961	0.33%	1961	0.26%
Inland marshes	324	0.05%	324	0.04%
Permanently irrigated land	311,240	51.94%	145,963	19.24%
Total “turtle habitat”	313,525	52.32%	148,247	19.54%
Total area	599,236		758,592	

## 4 Discussion

Here we mainly focused on recent changes in sand occurrences in the areas along the upper part of the Tigris possibly accessible to the Euphrates Softshell Turtle, assuming sandbanks as a limiting factor for nesting. We considered any remotely detectable amount of vegetation as unfavorable/unsuitable areas for nesting of the species. Although Ghaffari et al. (2013) reported a relatively high (44%) vegetation density at immediate vicinity of the single nest found in Iran, we assume that this would be more appropriate for tributaries such as in the case reported, and might not be applicable to the main channel of the river in our study area, where natural vegetation is relatively sparse (Biricik and Turğa 2011). Additionally, climatic differences between the two nesting sites mentioned, as a result of the latitudinal difference of 5.45° (> 600 km, locality in Iran being further south), should be taken into consideration. The river surface in the vicinity of the nest site in Iran experienced extreme heat (49°C) at the time of hatchling emergence, and vegetation cover surrounding the nest typically lower the nest temperature and could affect egg and hatchling survival.

At this point, it might be noteworthy to add that dams and irrigation projects may facilitate the spread of the tamarisk (*Tamarix* spp.) by creating large‐scale changes in river systems (Nilsson and Berggren 2000). This transformation as an indirect impact also seems to apply to the Tigris area, which could result in large areas becoming inaccessible to the species.

Prediction maps of our ensemble model for 2018 and 2022 suggest that suitable nesting habitat quality was generally low in both periods (Figure 4). The model predicted 95.2% of the areas as “low suitability” (cell classes 1–3) in 2018, a value further rising to 98.0% in 2022. The results concluded that about 59% of the cells (417 out of 703) that were considered resembling relatively “good suitability” (classes 5–6) have been lost within 4 years (Table 3, Figure 4). The distribution of the few raster map cells with higher sand probability values remaining shows very high fragmentation over a larger shoreline zone (Figure 5). This would mean lower accessibility and further lowered reproductive success for the species.

Despite our limited knowledge on the reproductive biology of the Euphrates Softshell Turtle (Biricik and Turğa 2011; Ghaffari et al. 2013; Bayrakçı 2023), their need for sandy nest sites is evident, and the deficiency of suitable sandy areas, preeminently sandbars, is likely to seriously impact the survival of the species. Sandbars are essential to the life cycle of many wildlife species: Being free of vegetation, sandbars are exposed to full sunlight, so they are vital for reptiles that need warmth for their eggs to hatch. Sandbar soil texture affects temperature which plays a central role in turtle egg development (Lenhart, Naber, and Nieber 2013). In reptiles, variable developmental environments influence gas and water exchange and the temperature experienced by the eggs; during incubation, these micro‐environments may influence developmental rate, offspring size, locomotor speed, thermoregulation behavior, and growth rate (Kolbe and Janzen 2002). Turtles use a variety of environmental cues to select a nest site such as slope, temperature, distance from water, substrate composition, moisture content, compactness, pH, salinity, and vegetation cover (Wyneken, Godfrey, and Bels 2008). In the absence of adequate nesting habitat, nest‐site selection by females may be compromised as nests may be placed even if they may not represent a good choice, and thus negatively impact egg development or the hatchlings (Bárcenas‐García et al. 2022). Habitat modification lowering nest success despite adaptive nesting behavior could constitute an ecological trap. Such ecological traps arise when human modifications of the habitat occur at a rate faster than the populations can respond locking such populations in the inability to cope with the altered habitat (Kolbe and Janzen 2002). On the other hand, declines in offspring numbers and reproductive output in turtles are notoriously hard to detect due to their long life‐spans and low adult mortality (Moll and Moll 2004; Stanford et al. 2020). The correlation between reduction in nesting sites availability and population levels is complex and includes a host of natural and anthropogenic factors (Lenhart, Naber, and Nieber 2013). Yet, we can conclude that the stability of the Tigris population of the species in Turkey is likely to be highly sensitive to changes in egg survival and hatching success.

Bayrakçı, Ayaz, and Luiselli (2021) evaluated the habitat change in the Euphrates between the 1990s and 2018, and concluded that the areas potentially available to the Euphrates Softshell Turtle increased by 413%. Although the authors stated that an increase of water bodies had no contribution to the habitats of *R. euphraticus* and that large reservoirs had long shorelines but lacked native bank vegetation, and are thus unfavorable for the species, they did not clearly differentiate between “*available/turtle habitat*” and “*nesting habitat*”. The authors included permanently irrigated land as a “preferred turtle habitat”, assuming that turtles, once forced, would consequently transition to oviposit in these areas owing to alteration of their most suitable habitat. “Turtle nests and live individuals observed at permanently irrigated land sites” were the basis for this speculation, however without any further information on the nests mentioned. We may disagree with this conjecture unreservedly, not only due to the lack of supporting data but also based on the presumption that any nesting attempt on irrigated farmlands would have very small chances to result in successful breeding mainly owing to the big differences in micro‐climatic conditions in nest chambers (Mortimer 1990). Additionally, we hypothesize that the siltation caused by artificially changed water regimes (Stanford et al. 2020) might be an additional damaging process in riverbanks of the Tigris as a result of irrigation projects, begging further thorough investigation. Sticky particles in lower size would clog egg pores, thus prevent adequate gas exchange between the embryo and the environment (Roosenburg and Place 1995). However, it must be emphasized again how inadequate our current knowledge is and that we have not yet understood how flexible the species can actually be in its choice of nesting site and to what extent it could successfully use available suboptimal areas.

Apart from the general regulations for wildlife, there is currently no conservation program for the Tigris populations of the Euphrates Softshell Turtle. The Turkish Ministry of Forestry and Water Management had prepared a five‐year action plan for the species (OSB 2015). The plan aimed to “protect the habitats and keep the existing population of the Euphrates Softshell Turtle in ecological balance, by raising awareness”. However, these plans were restricted to a small population remaining between the embankments of Birecik and Karkamış Dams in the Euphrates area (*see* Figure 2), where four nests were detected (within about 140 m distance) at a later time by Bayrakçı (2023). Several action items were suggested in the plan including (i) conservation and management of the habitats by means of the creation of new protected areas, (ii) scientific research and monitoring, (iii) raising awareness, capacity building, and training activities, and (iv) periodic reassessment of the species action plan. To protect the existing habitats, it was intended not to renew the licenses of sand quarries operating in the Euphrates, nor to issue operating permits in new areas. However, some of the latest local news from the area reveal that sand mining activities are still ongoing. It appears that the other goals of the plan, with some exceptions, such as radio‐tracking of individuals in order to detect their home ranges (Bayrakçı, Ayaz, and Luiselli 2021), were also largely not to be achieved.

Actions are however urgently required to prevent the very imaginable and imminent collapse of existing populations of the species in the Tigris area. Among the measures to mitigate dam impacts on freshwater turtles, the most frequently suggested were and still are habitat conservation, flow regulation, and environmental education (Ghaffari et al. 2014; Bárcenas‐García et al. 2022). Promoting habitat creation and restoration, which should be straight forward to implement, would likely contribute to long‐term conservation of nesting areas for turtles. However, currently no studies exist that evaluate the implementation of these suggested measures for freshwater turtle species impacted by dams (Bárcenas‐García et al. 2022). In any case, preventing the extinction of the local population in Turkey requires strictly protecting remaining habitat. Along the waterway, development should be strongly restricted to protect from further disturbances and encroachment. Environmental authorities must conduct more robust and rigorous environmental impact assessments, as well as provide support to supervise compliance with mitigation actions and monitoring effectiveness (Bárcenas‐García et al. 2022). It is necessary to strengthen the protection and long‐term monitoring of existing nesting areas through scientific research. There is a need to develop integrated and adaptive approaches, such as community‐based conservation (Berkes 2004), which requires active involvement of the communities in creating plans and/or management projects, as well as the promotion of social, governmental, economic, and academic participation. Translocations as a form of rescue from areas particularly susceptible by habitat devastation could support existing populations in better suited habitats or serve the creation of self‐sustaining populations elsewhere. And finally, should in situ conservation efforts fail, assurance colonies for potential reintroductions should be created by captive breeding program to maintain this unique natural heritage.

## Author Contributions

**Murat Biricik:** conceptualization (equal), data curation (equal), formal analysis (equal), funding acquisition (equal), investigation (equal), methodology (equal), project administration (equal), writing – original draft (equal), writing – review and editing (equal). **Kamran Safi:** conceptualization (equal), formal analysis (equal), funding acquisition (equal), methodology (equal), project administration (equal), supervision (lead), writing – original draft (equal), writing – review and editing (equal). **Şemsettin Turğa:** conceptualization (equal), data curation (equal), investigation (equal), writing – original draft (equal).

## Conflicts of Interest

The authors declare no conflicts of interest.

## Supporting information


**Table S1.** Presence and absence (occ = 1/0) point coordinates (in decimal degrees) used in this study.

**Figure S1.** Average response curves of variables in the single models.

**Figure S2.** Average importance of variables in the single models.

## Data Availability

All code and data used in this study are available at https://edmond.mpg.de/dataset.xhtml?persistentId=doi:10.17617/3.ULFA7J.
