# Supplementary figures and images for: Remote Sensing Data Reveal a Significant Reduction in the Area of the Nesting Habitat of Rafetus euphraticus in the Tigris River, Southeastern Turkey
Source: Ecol Evol. 2024 Dec 16;14(12):e70691. doi: 10.1002/ece3.70691 (PMC11650743; doi:10.1002/ece3.70691)

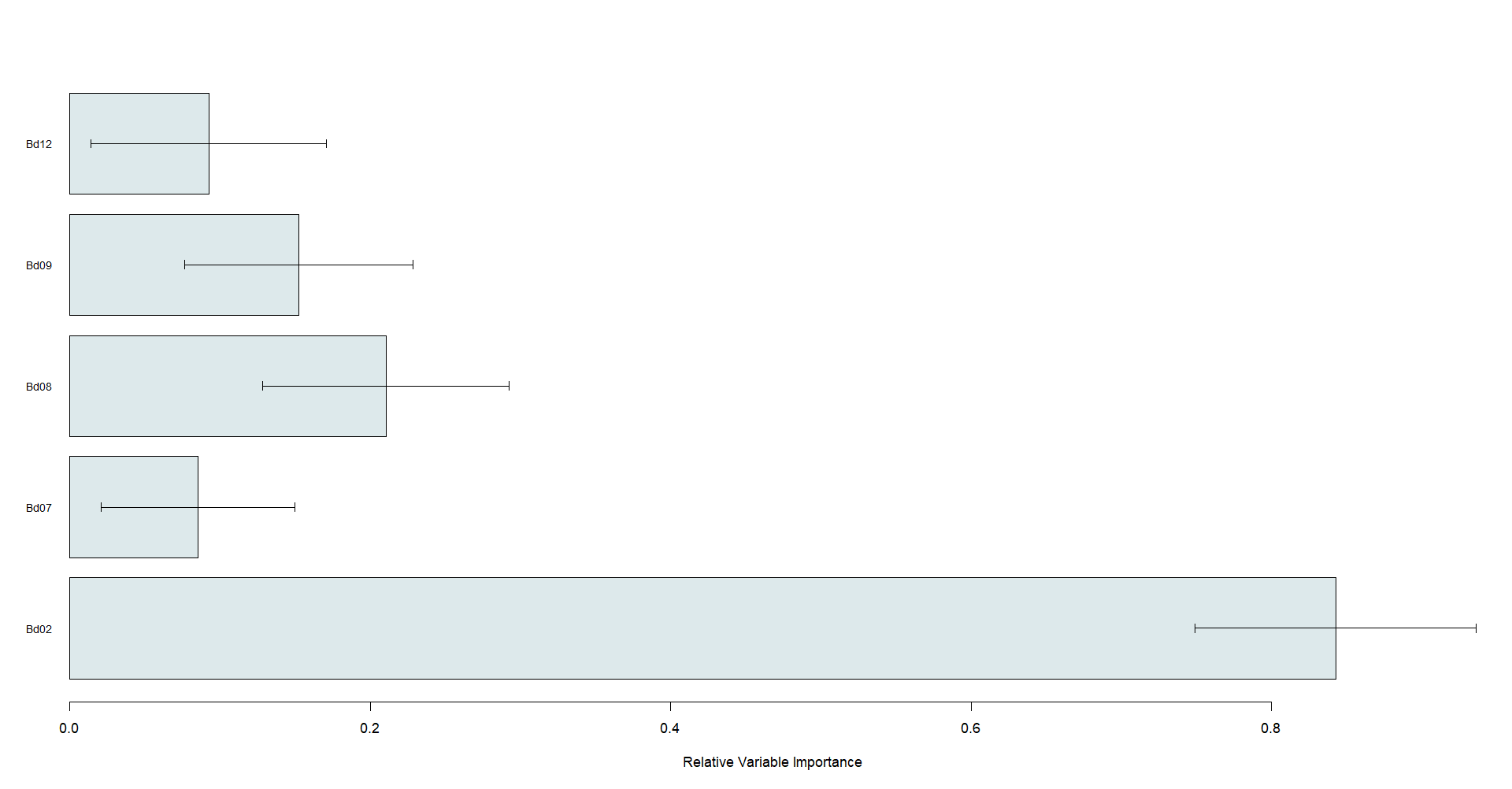

Supplement: Supplementary file 2 — Figure S1. Average response curves of variables in the single models. [file ECE3-14-e70691-s002.png]

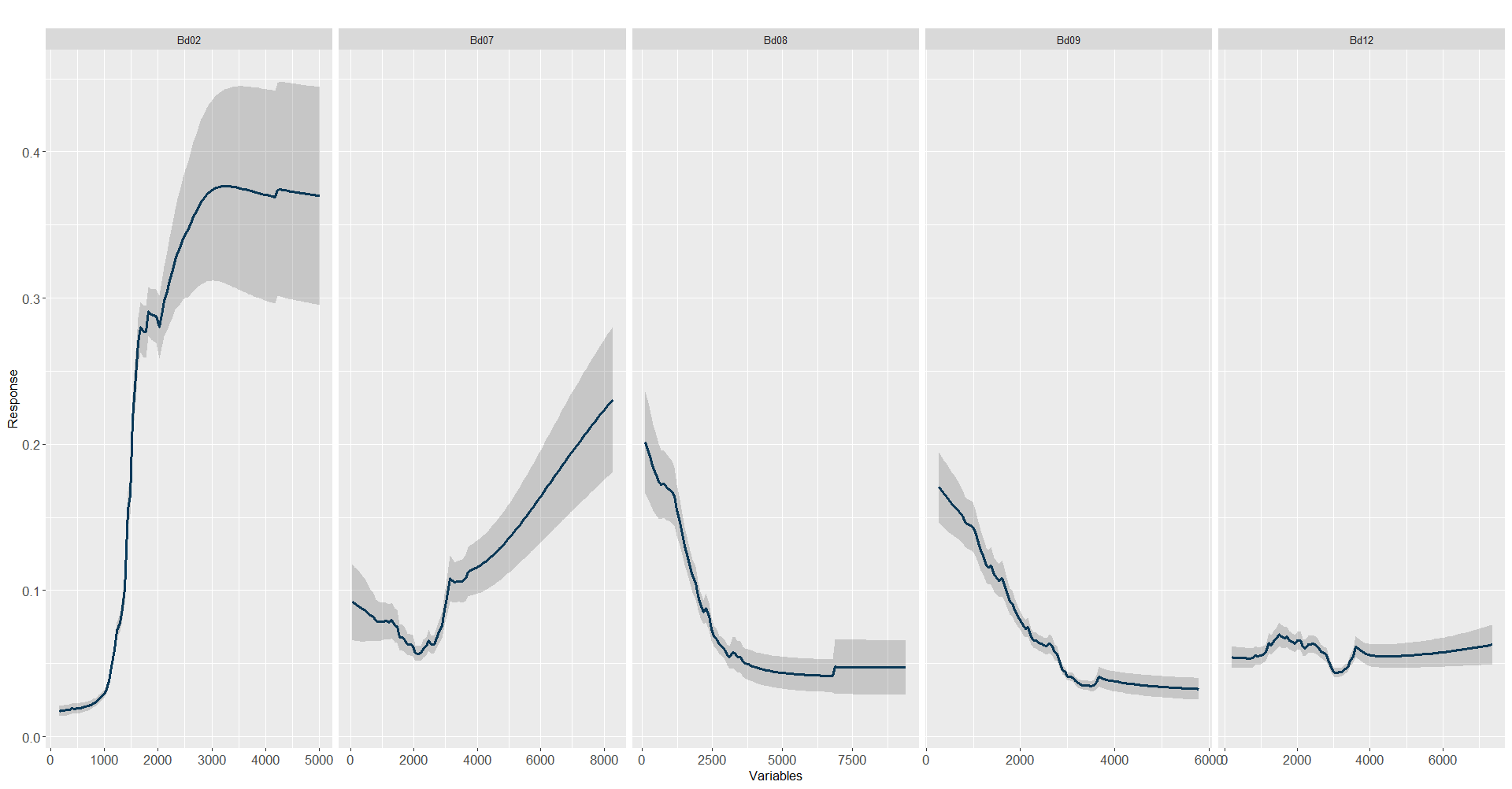

Supplement: Supplementary file 3 — Figure S2. Average importance of variables in the single models. [file ECE3-14-e70691-s001.png]
